# Improving cellulase production in submerged fermentation by the expression of a *Vitreoscilla* hemoglobin in *Trichoderma reesei*

**DOI:** 10.1186/s13568-017-0507-x

**Published:** 2017-11-15

**Authors:** Jie Lin, Xiamei Zhang, Bingran Song, Wei Xue, Xiaoyun Su, Xiuzhen Chen, Zhiyang Dong

**Affiliations:** 1 Institute of Microbiology, Chinese Academy of Sciences, No. 1 West Beichen Road, Chaoyang District, Beijing, 100101 People’s Republic of China; 2 University of Chinese Academy of Sciences, Beijing, 100049 People’s Republic of China; 3 Key Laboratory for Feed Biotechnology of the Ministry of Agriculture, Feed Research Institute, Chinese Academy of Agricultural Sciences, Beijing, 100081 People’s Republic of China

**Keywords:** *Trichoderma reesei*, *Vitreoscilla* hemoglobin, Submerged fermentation, Oxygen limitation, Cellulase

## Abstract

*Trichoderma reesei* is well known as an industrial workhorse fungus in cellulase production. The low dissolved oxygen supply in the highly viscous medium of *T. reesei* remains a major bottleneck that hampers growth and cellulase production in submerged fermentation. *Vitreoscilla* hemoglobin (VHb) has been demonstrated to improve metabolism and protein production in different heterologous hosts under hypoxic conditions, but the use of VHb in *T. reesei* remains uninvestigated. This study examines the effect of VHb in improving *T. reesei* performance in submerged fermentation. The VHb gene (*vgb*)-expressing cassette was successfully transformed into the TU-6 strain, integrated into the genome of *T. reesei*, and functionally expressed with biological activity, which was confirmed by carbon monoxide difference analysis. Compared to the parent strain, the expression of VHb increased the glucose consumption rate of the transformant. Moreover, in cellulase-inducing medium total protein secretion of the VHb expressing strain was 2.2-fold of the parental strain and the filter paper cellulase activity was increased by 58% under oxygen-limiting conditions. In summary, our results demonstrate that VHb has beneficial effects on improving total protein secretion and cellulase activity of *T. reesei* in submerged fermentation.

**Electronic supplementary material:**

The online version of this article (10.1186/s13568-017-0507-x) contains supplementary material, which is available to authorized users.

## Introduction

Lignocellulosic biomass is the most abundant renewable resource on Earth. The bioconversion of lignocellulose into liquid biofuels and biochemicals using enzymatic depolymerization with subsequent microbial fermentation is an important strategy in dealing with the global energy shortage as well as associated environmental problems (Gupta and Verma 2015). Cellulase can efficiently degrade the complex polysaccharide components of lignocellulosic into monomeric sugars, which is a key process in biomass bioconversion (Sun and Cheng 2002). The high cost of cellulase production, however, remains a major obstacle that hinders scale-up of biotransformation of lignocellulose (Klein-Marcuschamer et al. 2012). Strategies that can improve cellulase production will be of great value for the efficient utilization of lignocellulose and the reduction of cellulase cost.

*Trichoderma reesei* (teleomorph *Hypocrea jecorina*) is one of the most important commercial cellulase producers and has been widely used in a variety of industries, including food, feed and biorefinery (Bouws et al. 2008; Schuster and Schmoll 2010). In industry, cellulase is produced by *T. reesei* mainly by way of submerged fermentation, which is an aerobic fermentation process with a long culture period. Thus, adequate dissolved oxygen is required to maintain cell growth and metabolism during the liquid-state fermentation. However, the high viscosity of the cultures severely hinders mass mixing and oxygen transfer, which directly leads to metabolic activity slowdown, cell growth retardation and even cell death. Consequently, fermentation production of cellulase is hampered (Peciulyte et al. 2014). Traditional approaches such as increasing agitation rate or ventilation to enhance oxygen supply are costly and often have little effect on ameliorating the oxygen limitation (Serrano-Carreon et al. 2015).

The bacterial hemoglobin (VHb) from the obligate aerobic bacterium *Vitreoscilla* is an oxygen-binding protein, functioning as an oxygen carrier and transporter (Wakabayashi et al. 1986). In bacteria (Horng et al. 2010), yeasts (Wu and Fu 2012), plants (Jokipii et al. 2008) and animals (Pendse and Bailey 1994), the introduction of VHb has been proven to efficiently facilitate cellular aerobic metabolism, by which the growth rate and protein synthesis are improved under oxygen-limited conditions. Recently, examples have demonstrated that this strategy also works well in filamentous fungi. For example, VHb expression increased the yields of biomass, protease and exopectinases of *Aspergillus sojae* in solid-state fermentation, in which strains encounter oxygen transfer problems (Mora-Lugo et al. 2015). *Paecilomyces lilacinus* carrying the *vgb* gene has also been shown to yield higher amounts of biomass, protease and chitinase under hypoxic conditions during submerged fermentation, which improves the value of this biocontrol fungus in practical applications (Zhang et al. 2014). In *Aspergillus niger*, secreted metabolites and oxygen uptake were analyzed and demonstrated that VHb expression technology is an effective strategy to reduce unwanted side effects of oxygen limitation during submerged fermentation, and was particularly beneficial to filamentous fungi where oxygen transfer to the cell is often limited by the highly viscous broth (Hofmann et al. 2009). However, this strategy has not been explored in the industrial strain *T. reesei* until now.

In this study, we report for the first time application of the VHb expression technology in *T. reesei.* The VHb gene (*vgb*) was successfully integrated into the *T. reesei* genome, and the effects of VHb expression on the growth, total protein secretion and production of cellulase were analyzed. Our study provides an efficient means to improve the endogenous cellulase production of *T. reesei*, and sheds light on improving exogenous protein expression.

## Materials and methods

### Strains and media

The *T. reesei* TU-6 strain (ATCC MYA-256, a uridine auxotrophic strain) was grown at 28 °C for 7–10 days on potato dextrose agar (PDA) plates supplemented with 5 mM uridine for sporulation. Minimal medium (Pentillä et al. 1987) was supplemented with 2% glucose or 1% Avicel PH101 (Sigma-Aldrich, St. Louis, MO) as the sole carbon source and used for fungal vegetative growth and fermentation, respectively. When necessary, 5 mM uridine was added to the culture medium for TU-6. All DNA manipulations were carried out in *Escherichia coli* strain DH5α (TransGen Biotech, Beijing, China).

### Plasmid construction and fungal transformation

The plasmids pUCVHb and pNOM102 (Roberts et al. 1989) were kindly provided by Professor Guomin Tang from the Institute of Microbiology, Chinese Academy of Sciences. The *vgb* gene (GenBank accession number: L21670) encoding the *Vitreoscilla* hemoglobin protein was cloned from the pUCVHb plasmid with the primers VHb-F and VHb-R (Table 1). The pNOM102 vector backbone, which contained the strong constitutive *Aspergillus nidulans gpd*A promoter (P*gpd*A) and *trp*C terminator (T*trp*C), was amplified with the primers TRP-F and GPD-R (Table 1). The plasmid pNOM102-VHb was constructed by placing the *vgb* gene under the control of the *gpd*A promoter and *trp*C terminator with a MultiS One-Step Cloning Kit (Vazyme Biotech, Nanjing, China).

**Table 1:** Primers used in this study

Primer	Primer sequence (5′–3′)
VHb-F	AGACATCACAATGTTAGACCAGCAAACCAT
VHb-R	TTAATGATGATGATGATGATGTTCAACCGCTTGAGCGTA
TRP-F	ATCATCATCATCATCATTAAGGATCCACTTAACGTTACTG
GPD-R	GGTCTAACATTGTGATGTCTGCTCAAGCGG
TF	AAGGATTTCGGCACGGCTAC
TR	GCACTCTTTGCTGCTTGGAC
M13F	GTAAAACGACGGCCAGT
M13R	CAGGAAACAGCTATGACC

The underlined nucleotide sequence represents the C-terminal 6×His tag

**Fig. 1:**
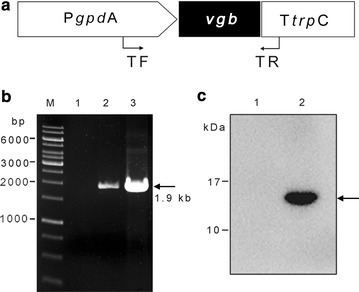
Identification of the TU6-vgb^+^ transformant. **a** The *vgb*-expressing cassette from the plasmid pNOM102-VHb. TF and TR indicate the two primers used for PCR verification; **b** identification of the putative *vgb* transformant with PCR analysis. PCR products with the expected size (1.9 kb) are indicated by an arrow. Genomic DNA from the parental *T. reesei* strain TU-6 (lane 1), the transformant (lane 2) and vector DNA from pNOM102-VHb (lane 3) were used as DNA templates; **c** Western-blot analysis of the intracellular protein extracts from *T. reesei* after 48 h of growth in MM-glucose. Equal amounts of total protein were loaded in lanes 1–2. A band with the expected size (16 kDa) are indicated by an arrow. Lane 1 represents the parent strain TU-6 and lane 2 represents TU6-vgb^+^

The *vgb*-expression cassette (Fig. 1a) was amplified with the primers M13F and M13R (Table 1) from pNOM102-VHb and co-transformed into the TU-6 strain with the plasmid pSK-pyr4 (Qin et al. 2012) via the PEG-mediated protoplast transformation method (Pentillä et al. 1987). Transformants were selected on an MM (minimal medium) plate without uridine.

### Analysis of fungal transformants

To confirm the chromosomal integration of the heterologous *vgb* gene, genomic DNA from both the parental TU-6 strain and the transformants were isolated and used as templates. PCR analysis was performed to amplify the *vgb*-expression cassette with the specific primer pairs TF/TR (Table 1).

For western blot analysis, the TU-6 and the verified TU6-vgb^+^ strains were grown in glucose medium for 48 h at 28 °C at 200 rpm. Mycelia were then collected and washed twice with sterilized water for protein extraction. Equal amounts of mycelia were ground into powder with liquid nitrogen, homogenized in 50 mM potassium phosphate buffer (pH 7.0) and centrifuged at 4 °C at 5000*g* for 20 min. The supernatant was carefully collected for western blotting. The VHb protein was designed with a C-terminal 6×His tag, and its presence was verified by an anti-His mouse monoclonal antibody (TransGen Biotech, Beijing, China) according to the manufacturer’s instructions.

### CO-difference spectral assay of VHb

The activity of the expressed VHb was detected using the carbon monoxide difference spectral assay as described by Liu and Webster (1974). One milliliter of whole mycelia extracts from the TU-6 or TU6-vgb^+^ transformants was treated with 20 mg of sodium sulfite. The samples were then divided into two aliquots, one of which was bubbled with CO for 5 min, while the other was bubbled with air. Then, the samples were scanned in the 400–500 nm range with a UV-2450 spectrophotometer (Shimadzu, Shimane, Japan).

### Analysis of glucose consumption, extracellular protein and enzyme activity in shake-flask fermentation

To investigate the effects of VHb expression on the growth of *T. reesei*, equal amounts of spores from TU-6 and the TU6-vgb^+^ strain were inoculated into 1 l flasks containing 400 ml of MM-glucose and cultivated for 3 days at 28 °C by shaking at 200 rpm. The residual glucose concentration of the culture medium during growth was determined using a modified glucose oxidase method (Trinder 1969). The dry weight of the harvested mycelia was measured according to a previously described protocol (Aro et al. 2003). Microscopic images were captured on a Leica DM500 optical microscope (Leica, Wetzlar, Germany).

For cellulase induction, mycelia were pre-cultured in MM-glucose for 36–48 h and were collected and washed twice with carbon-free medium. Mycelia weights of 1.0, 1.6 and 2 g were transferred to 50, 80 and 100 ml of liquid medium with 1% Avicel cellulose, respectively, and grown for 144 h at 200 rpm at 28 °C (Zhang et al. 2012). The biomass in cellulose-containing medium was represented by protein concentration and was measured according to the method as described by Schuster et al. (2011). The extracellular protein concentration was measured using a Modified Bradford Protein Assay Kit (Sangon, Shanghai, China). For the cellulase activity assay, the filter paper activity (FPA) and the carboxymethylcellulose (CMCase) activity were measured according to IUPAC methods (Ghose 1987).

## Results

### Verification of *vgb* expression in the *T. reesei* TU6-vgb^+^ transformant

The plasmid pNOM102-VHb contained the *vgb* gene under the control of the *gpd*A promoter and *trp*C terminator and was co-transformed into the *T. reesei* TU-6 strain with the plasmid pSK-pyr4, which was carrying the *pyr4* gene to complement the uridine auxotroph. To determine integration of the *vgb* gene into the *T. reesei* genome, PCR was performed using the genomic DNA isolated from TU-6 and the selected transformants. Twenty-one out of 23 transformants were confirmed containing the *vgb* gene (data not shown). Figure 1b showed that the band corresponding to the expected size (1.9 kb) of the *vgb* expression cassette was present in the transformant but absent in the TU-6 strain. Sequencing of the PCR product result further confirmed that the *vgb*-expression cassette was successfully integrated into the *T. reesei* genome.

It has been reported that there are often difficulties in expressing bacterial proteins in filamentous fungi (Su et al. 2012). Therefore, western blotting was performed to verify whether VHb was successfully expressed. As shown in Fig. 1c, a band with an approximate size of 16 kDa, corresponding to the calculated molecular mass of VHb, was detected in the TU6-vgb^+^ transformant but absent in the parental strain, indicating that *T. reesei* successfully expressed the VHb protein.

### CO-difference spectral analysis

**Fig. 2:**
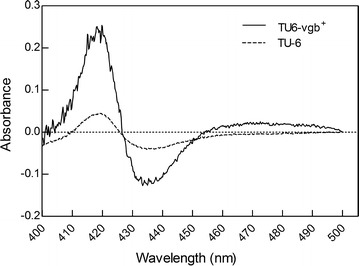
CO-difference spectral analysis of *T. reesei* TU-6 and TU6-vgb^+^. CO-difference spectra of mycelia extracts were measured between 400 and 500 nm after bubbling with CO. TU6-vgb^+^ is shown with a black solid line and TU-6 is shown with a dashed line

The activity of the expressed VHb protein in *T. reesei* was determined by CO-difference spectral analysis as described by Liu and Webster (1974). The *Vitreoscilla* hemoglobin (VHb) has a specific absorption peak at 420 nm. As expected, when the mycelia extract of the VHb transformant (TU6-vgb^+^) was bubbled with CO, it showed a characteristic VHb CO-binding absorbance peak at 420 nm (Fig. 2), indicating that the expressed VHb protein in *T. reesei* retained the biological function of hemoglobin. The small peak observed in the parental strain might be the result of other CO-binding proteins.

### VHb expression improve glucose consumption rate of *T. reesei*

**Fig. 3:**
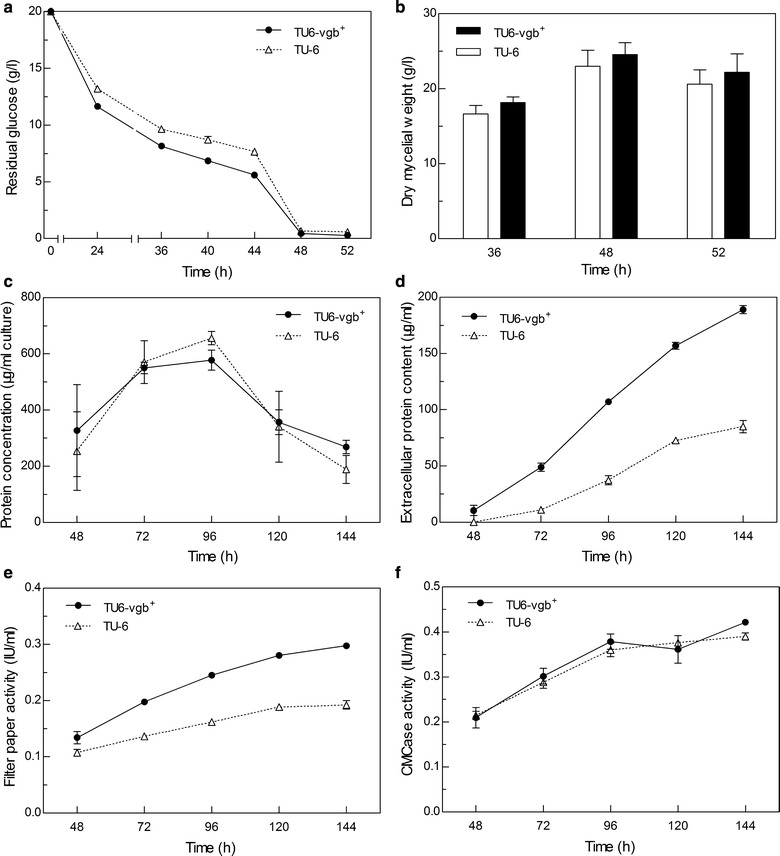
Growth and protein production of TU-6 and TU6-vgb^+^ in batch cultures. Glucose concentration (**a**) in the culture medium and dry mycelia weight (**b**) were measured at different time points for strains cultured in MM-glucose. In cellulose-containing medium the biomass (**c**) which was represented by protein content, the extracellular protein concentration (**d**), FPase activity (**e**) and CMCase activity (**f**) were measured under oxygen-limiting conditions. TU6-vgb^+^ is shown as black solid lines or black bars and TU-6 is shown as dashed lines or white bars. Data represented are the means of three independent cultures

The rates of glucose consumption and dry mycelia weights were measured to assess the growth rates of *T. reesei* strains. Figure 3a shows that the glucose consumption rate of TU6-vgb^+^ was much higher than that of the TU-6 strain in glucose-containing medium, especially during the time period from 36 h to 44 h. However, the dry mycelia weight of TU6-vgb^+^ showed no significant increase in comparison to TU-6 strain (Fig. 3b). In addition, unlike the parental strain which was prone to form lots of irregular aggregated hyphal pellets, the mycelia of TU6-vgb^+^ were uniform and dispersive with only a few pellets, but there was no obvious morphological difference under microscopic observation (Additional file 1: Figure S1).

### VHb expression has an improved effect on cellulase production as culture volumes increased

The influence of culture medium volume on *T. reesei* cellulase production was investigated. Strains were cultivated in the same size flask with different volumes (50, 80 and 100 ml) of cellulase-inducing medium. As shown in Table 2, TU-6 and TU6-vgb^+^ both produced less cellulase after 144 h cultivation as the inductive fermentation volume increased. Moreover, the FPase activity of TU6-vgb^+^ increased by 21% in 80 ml culture medium and increased by 58% in 100 ml culture medium compared with the parental TU-6 strain, although no pronounced difference of FPase activity was observed when strains were cultivated in 50 ml culture medium. This clearly demonstrates the positive effects of VHb expression on cellulase production for cultures with decreased dissolved oxygen.

**Table 2:** FPase activities of *T. reesei* TU-6 and TU6-vgb^+^ in different culture volumes

Culture volume (ml)/250 ml	FPU		Increasing rate
	TU-6	TU6-vgb^+^	
50	0.78 (± 0.036)	0.78 (± 0.062)	0
80	0.43 (± 0.131)	0.52 (± 0.063)	21%
100	0.19 (± 0.013)	0.30 (± 0.001)	58%

All cultures were grown in 250 ml flasks with different volumes (50, 80 and 100 ml) of cellulase-inducing medium. Data represented are the means of three independent cultures, SD are given in brackets

### VHb expression increases extracellular protein secretion and cellulase production in 100 ml culture medium

**Fig. 4:**
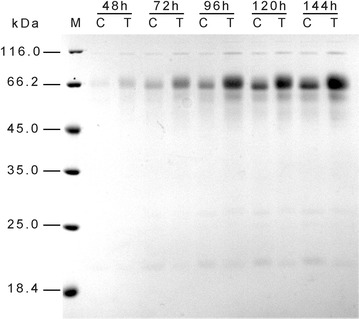
SDS-PAGE analysis of total extracellular protein in culture supernatants. TU-6 (C, indicating control) and VHb transformant TU6-vgb^+^ (T, meaning test) were grown in 100 ml cellulase-inducing medium for 144 h. Equal amounts of culture supernatants were loaded per lane. The fermentations were carried out in triplicates

To examine the effects of VHb on the production of extracellular protein and cellulase, TU-6 and three *vgb* transformants (VHb1, VHb2 and VHb3) were cultivated in 100 ml cellulase-inducing medium, and fermentation samples were collected at indicated time points and analyzed for biomass, protein concentration and enzymatic activities (Fig. 3c–f and Additional file 1: Figure S2). Compared with the parental strain, the biomass of TU6-vgb^+^ showed no significant increase during the cultivation as shown in Fig. 3c. However, the extracellular protein concentration in the TU6-vgb^+^ culture supernatant was significantly elevated after 72 h (Fig. 3d). The maximum extracellular protein concentration in the VHb-expressing strain was almost 2.2-fold of the parental strain. SDS-PAGE analysis confirmed the enhanced protein production in TU6-vgb^+^ (Fig. 4). Cellulase activities in the culture supernatant of the *vgb*-transformed strains were also increased. Compared to the parental strain, the maximum FPase activity of the TU6-vgb^+^ transformant significantly increased by 58% at 144 h (Fig. 3e). In contrast, the carboxymethylcellulose (CMCase) activity showed no obvious differences between the two strains (Fig. 3f). Extracellular protein concentration and the FPase activity of the other two transformants (VHb2 and VHb3) also showed a similar increase, as shown in Additional file 1: Figure S2.

## Discussion

*Trichoderma reesei* has been widely used for the production of cellulase (Zhang et al. 2017). However, poor oxygen transfer in the highly viscous medium of this filamentous fungi remains one of the major challenges hampering cellulase production in submerged fermentation. Previous studies have shown that the hemoglobin protein VHb from *Vitreoscilla* sp. could efficiently alleviate oxygen-limiting conditions and improve cell growth, protein synthesis and metabolic productivity in various organisms, such as bacteria, fungi, plants and animals (Frey and Kallio 2003; Stark et al. 2015). It, however, has not been explored in the cellulase producer *T. reesei* until now. Thus, the aim of our study was to explore the role of VHb in alleviating oxygen limitation in *T. reesei* submerged fermentation, thereby improving cell growth and major cellulase production.

The expression of VHb facilitated the glucose consumption rate of *T. reesei*, which coincides with previously reported results in *A. niger* and *Bacillus subtilis* (Hofmann et al. 2009; Su et al. 2010). However, the VHb expression did not improved the growth (dry mycelia weight) of the transformants in glucose-containing medium, nor did it increase the biomass of TU6-vgb^+^ in the cellulose-containing medium. These results were consistent with the studies in *E. coli* (Geckil et al. 2001), *Serratia marcescens* (Wei et al. 1998) and *Ganoderma lucidum* (Li et al. 2016) that the VHb-expressing strains did not grow better than the strains without VHb, but contrary to the study in *A. sojae* (Mora-Lugo et al. 2015) and *P. lilacinus* (Zhang et al. 2014). It was perhaps that the effect of VHb on the biomass may vary in different organisms.

When the strains grew in glucose medium, the mycelia of TU6-vgb^+^ were uniform and dispersive with fewer pellets in the culture medium than TU-6. Previous reports have shown that the embedded hyphae in pellets are supplied with limited oxygen and nutrition, which subsequently impair growth of the hyphae and can even lead to cell autolysis (Papagianni 2004). In *Yarrowia lipolytica*, VHb expression has a beneficial influence on the morphology of the host cell, resulting in better utilization of available oxygen (Bhave and Chattoo 2003). Therefore, it is reasonable to speculate that the uniform and dispersive mycelia of the TU6-vgb^+^ transformants might be the result of the expressed VHb, and its resulting improved oxygen transfer in submerged fermentation.

The extracellular protein production and FPase activity also increased in the VHb expression transformants. Similar observations were also reported for *A. sojae* and *P. lilacinus* (Mora-Lugo et al. 2015; Zhang et al. 2014). Elevated protease and exo-pectinase content was observed when a *vgb* gene was engineered into *A. sojae*. Additionally, the expression of VHb in *P. lilacinus* improved protease and chitinase secretion under oxygen-limiting conditions. In summary, the protein production improvements of the *T. reesei* TU6-vgb^+^ transformants shown in our study demonstrated that VHb was conducive to improving the strain’s adaptability to oxygen-limiting conditions in viscous fermentation medium.

Contrary to the increased FPase activity, CMCase activity had no obvious enhancement in TU6-vgb^+^. A reasonable explanation is that FPase activity represents the synergistic cellulose hydrolytic activity of three cellulase categories, including endo-β-1,4-glucanases, cellobiohydrolases, and β-glucosidases, whereas CMCase activity merely represents the activity of endo-β-1,4-glucanases. The expression of VHb might have improved FPase activity by enhancing the expression of parts of the overall cellulase activity such as cellobiohydrolases (CBH1, CBH2) and β-glucosidases (BGL1, BGL2), but not endo-β-1,4-glucanases (EG1-5). Similar results that VHb expression did not equally influence the expression of all native genes were also reported in *E. coli* and *A. sojae* (Mora-Lugo et al. 2015; Roos et al. 2004).

The fact that cellulase production decreased as culture medium volume increased in the same-sized flask further confirms that an insufficient amount of dissolved oxygen is an important factor that limits cellulase production in *T. reesei* submerged fermentation. The expression of VHb protein efficiently mitigated this limitation to some degree. This result was consistent with many studies that have been carried out in *Y. lipolytica*, *Pseudomonas* and *Burkholderia*, *Schwanniomyces occidentalis* and *P. lilacinus* (Bhave and Chattoo 2003; Kim et al. 2005; Suthar and Chattoo 2006; Zhang et al. 2014). In these microorganisms, the expression of VHb causes an increased enhancement of protein production under oxygen-limiting conditions as compared to normal conditions.

Even though the underlying mechanism explaining the beneficial effects of VHb on protein production has not been clearly established, various studies demonstrated that VHb might participate in one or more steps of the respiratory chain. VHb was first found to bind oxygen and deliver it to the respiratory apparatus under hypoxic conditions (Stark et al. 2011; Webster 1988). Then, it was reported that VHb can act as a terminal oxidase for facilitating ATP production (Dikshit et al. 1992) and can even take part in the regulation of host gene expression (Roos et al. 2004). Therefore, the beneficial effects of VHb expression on the growth and cellulase production of *T. reesei* might be the result of the combined functions of VHb.

This study is the first report of applying the VHb technology in the cellulase producer *T. reesei*. The successful expression of a functional VHb from *Vitreoscilla* sp. significantly increased total protein secretion and cellulase production of *T. reesei* during submerged fermentation under hypoxic conditions. The results clearly demonstrate that engineering a bacterial VHb into *T. reesei* is an effective strategy to improve cellulase production and may also provide an alternative method to improve heterologous protein expression in *T. reesei*.

## Additional file

**Additional file 1: Figure S1.** Morphology of TU-6 and TU6-vgb^+^ after growth in MM-glucose.

**Additional file 2: Figure S2.** Time course determination of the extracellular protein concentration (a) and FPase activity (b) in the VHb-expressing strains VHb2 and VHb3 in cellulase-inducing medium under oxygen-limiting conditions.

## Abbreviations

VHb: *Vitreoscilla* hemoglobin
*vgb*: *Vitreoscilla* hemoglobin gene
PDA: potato dextrose agar
PEG: polyethylene glycol
CO: carbon monoxide
MM: minimal medium
IUPAC: International Union of Pure and Applied Chemistry
FPU: filter paper unit
CMC: carboxymethylcellulose
SDS-PAGE: sodium dodecyl sulfate polyacrylamide gel electrophoresis
CBH: cellobiohydrolases
BGL: β-glucosidases
EG: endo-β-1,4-glucanases
ATP: adenosine triphosphate
