# Supplementary material for: Improving cellulase production in submerged fermentation by the expression of a Vitreoscilla hemoglobin in Trichoderma reesei
Source: AMB Express. 2017 Nov 15;7:203. doi: 10.1186/s13568-017-0507-x (PMC5688050; doi:10.1186/s13568-017-0507-x)
Supplement: Supplementary file 1 — Additional file 1: Figure S1. Morphology of TU-6 and TU6-vgb+ after growth in MM-glucose. [file 13568_2017_507_MOESM1_ESM.pdf]

Figure S1

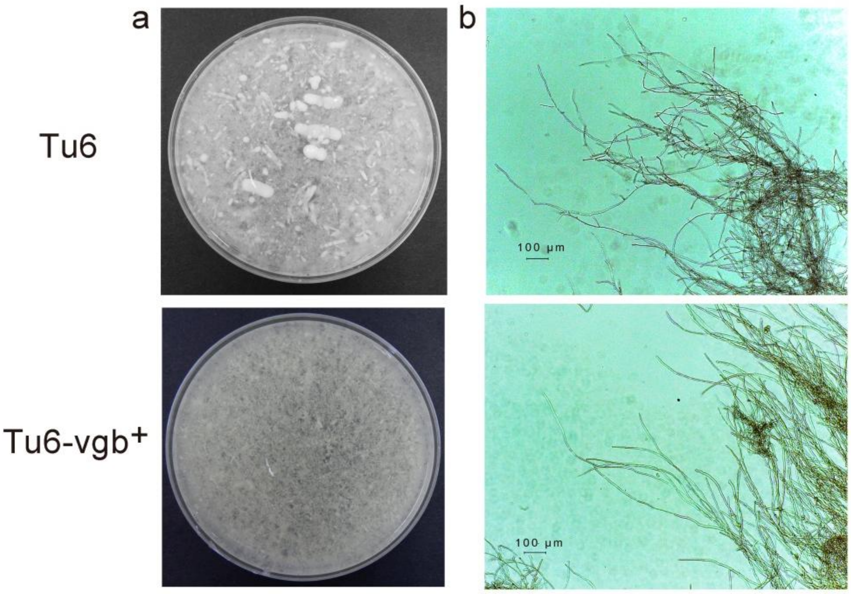

Figure S1 Morphology of TU-6 and TU6-vgb$^+$ after growth in MM-glucose. (**a**) The hyphal phenotypes of the two *T. reesei* strains cultivated in flasks as observed by the naked eye. (**b**) Microscopic observations of hyphae from TU-6 and TU6-vgb$^+$
