# Supplementary material for: Improving cellulase production in submerged fermentation by the expression of a Vitreoscilla hemoglobin in Trichoderma reesei
Source: AMB Express. 2017 Nov 15;7:203. doi: 10.1186/s13568-017-0507-x (PMC5688050; doi:10.1186/s13568-017-0507-x)
Supplement: Supplementary file 2 — Additional file 2: Figure S2. Time course determination of the extracellular protein concentration (a) and FPase activity (b) in the VHb-expressing strains VHb2 and VHb3 in cellulase-inducing medium under oxygen-limiting conditions. [file 13568_2017_507_MOESM2_ESM.pdf]

Figure S2

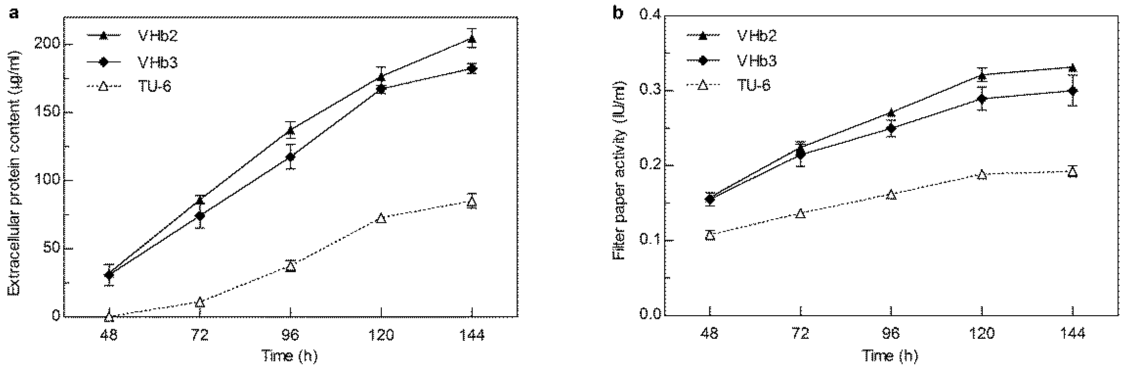

Figure S2 Time course determination of the extracellular protein concentration (**a**) and FPase activity (**b**) in the VHb-expressing strains VHb2 and VHb3 in cellulase-inducing medium under oxygen-limiting conditions. VHb2 (filled triangle) and VHb3(filled diamond) are shown as black solid lines and TU-6 (hollow triangle) is shown as dashed lines. Data represented are the means of three independent cultures
